# Heterologous expression of plant virus genes that suppress post-transcriptional gene silencing results in suppression of RNA interference in *Drosophila *cells

**DOI:** 10.1186/1472-6750-4-18

**Published:** 2004-08-25

**Authors:** Brian Reavy, Sheila Dawson, Tomas Canto, Stuart A MacFarlane

**Affiliations:** 1 Scottish Crop Research Institute, Invergowrie, Dundee DD2 5DA, UK

## Abstract

**Background:**

RNA interference (RNAi) in animals and post-transcriptional gene silencing (PTGS) in plants are related phenomena whose functions include the developmental regulation of gene expression and protection from transposable elements and viruses. Plant viruses respond by expressing suppressor proteins that interfere with the PTGS system.

**Results:**

Here we demonstrate that both transient and constitutive expression of the *Tobacco etch virus *HC-Pro silencing suppressor protein, which inhibits the maintenance of PTGS in plants, prevents dsRNA-induced RNAi of a *lac*Z gene in cultured *Drosophila *cells. Northern blot analysis of the RNA present in *Drosophila *cells showed that HC-Pro prevented degradation of *lacZ *RNA during RNAi but that there was accumulation of the short (23nt) RNA species associated with RNAi. A mutant HC-Pro that does not suppress PTGS in plants also does not affect RNAi in *Drosophila*. Similarly, the *Cucumber mosaic virus *2b protein, which inhibits the systemic spread of PTGS in plants, does not suppress RNAi in *Drosophila *cells. In addition, we have used the *Drosophila *system to demonstrate that the 16K cysteine-rich protein of *Tobacco rattle virus*, which previously had no known function, is a silencing suppressor protein.

**Conclusion:**

These results indicate that at least part of the process of RNAi in *Drosophila *and PTGS in plants is conserved, and that plant virus silencing suppressor proteins may be useful tools to investigate the mechanism of RNAi.

## Background

RNA interference (RNAi) is a process in which the introduction of dsRNAs into cells leads to inactivation of expression of genes containing homologous sequences by sequence-specific degradation of mRNA (for reviews see [1,2]). RNAi has been observed in a variety of organisms, including fruit fly, nematodes, zebrafish, mice and humans [3-7], and is mechanistically similar to post-transcriptional gene silencing (PTGS) in plants [8] and quelling in fungi [9]. Genetic studies have identified a number of the proteins that are involved in these processes in *Neurospora crassa *[10-12], *Caenorhabditis elegans *[13-15] and *Arabidopsis thaliana *[16-18]. dsRNA has been shown to induce RNAi in cultured *Drosophila *cells [19-22] and biochemical studies of this system have revealed the involvement of two distinct activities; an RNA-induced silencing complex (RISC), containing both nuclease activity and RNA that carries out enzymatic degradation of target RNA [19], and an RNase III-like protein (Dicer) that is involved in production of short (22 nucleotide) guide RNAs from dsRNA as an early step in the RNAi process [22].

PTGS in plants can operate as a defence mechanism against virus infection (for reviews see [8,23]) and numerous plant viruses encode silencing suppressors, which are thought to have developed as a response to the plant PTGS system [24]. Plant virus-encoded silencing suppressors may target different components of the PTGS system. The HC-Pro protein, encoded by potyviruses such as *Tobacco etch virus *(TEV), was initially characterised as a determinant of virus movement in plants [25] and subsequently was shown to inhibit silencing of transgenes in transformed plants [26]. HC-Pro antagonizes silencing in all tissues [26] and appears to target a step involved in the maintenance of silencing [27]. In contrast, the 2b protein encoded by *Cucumber mosaic virus *(CMV), a cucumovirus, interferes with the systemic spread of the silencing signal, preventing initiation of silencing in newly emerging tissues [28]. A survey of a small number of other different plant viruses showed that the comovirus *Cowpea mosaic virus*, the geminivirus *African cassava mosaic virus*, the potexvirus *Narcissus mosaic virus, *the tobamovirus TMV, the sobemovirus *Rice yellow mottle virus *(RYMV), the tombusvirus *Tomato bushy stunt virus *(TBSV) and the tobravirus *Tobacco rattle virus *(TRV) also were able to suppress GFP silencing [24]. Although in this study the potexvirus PVX did not suppress silencing, using a different assay these authors showed that PVX is in fact able to prevent systemic silencing [29]. Thus, it seems probable that many plant viruses encode proteins that allow them to evade or inhibit PTGS in certain plant species, and that different suppressor proteins target different parts of the PTGS pathway. An amenable system for studying these suppressor proteins would be an aid in determining the molecular basis of their action. Recently it has been demonstrated that an insect virus, flock house virus (FHV) encodes a protein, 2b, which acts as a suppressor of PTGS in plants [30]. We have investigated the effects on RNAi in cultured *Drosophila *cells of expression of some silencing suppressors from plant viruses.

## Results

### Effects of transient expression of the TEV HC-Pro and CMV 2b proteins on RNAi in *Drosophila *cells

**Figure 1:**
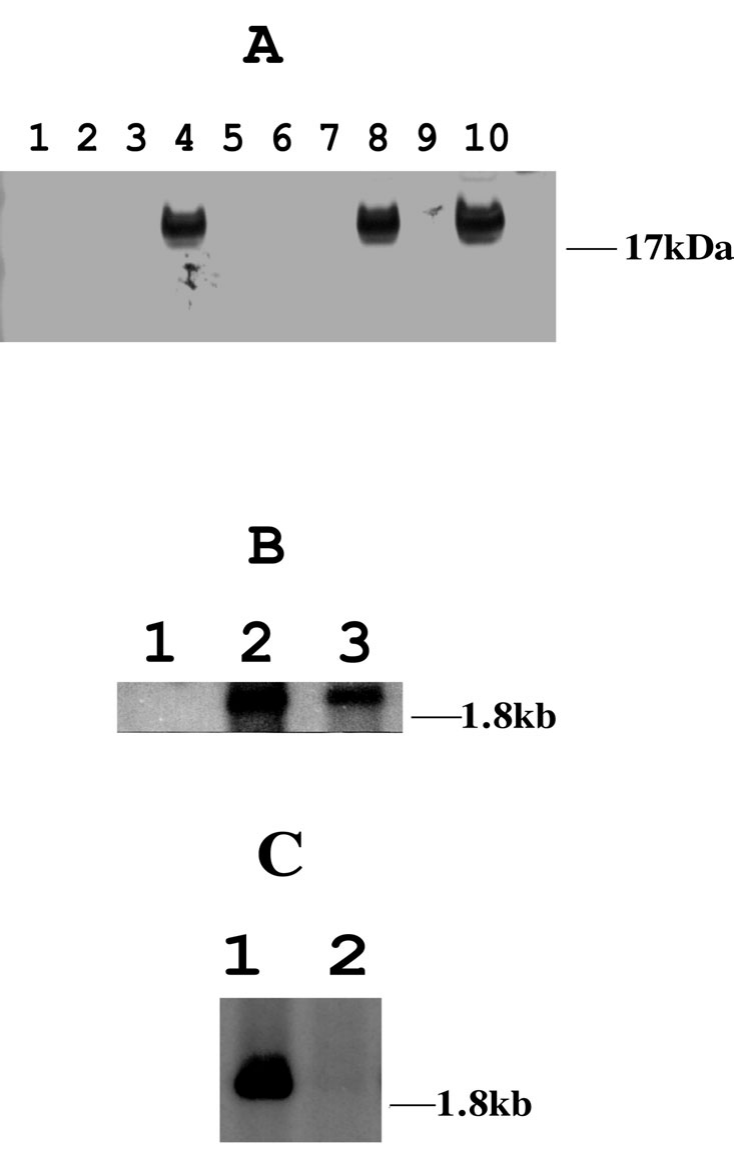
**Expression of plant virus silencing suppressor genes in *Drosophila *cells. **A. Immunoblot of lysates from control cells (lanes 1,2 and 5,6), cells transiently transfected with pMT-2b (lanes 3,4) or cells stably transformed with pMT-2b (lanes 7,8). Cells represented in lanes 2, 4, 6 and 8 were induced by addition of CuSO_4 _and cells represented in lanes 1, 3, 5 and 7 were uninduced. Lanes 9 and 10 were lysates from control plant or plant infected with CMV respectively. Lysates were analysed by polyacrylamide gel electrophoresis, proteins transferred to nitrocellulose by electroblotting and probed with an anti-2b antibody. The position of migration of a SeeBlue Plus 2 Pre-stained molecular weight marker (Invitrogen) band is shown. B. Northern blot analysis of RNA from control cells (lane 1) or cells transfected with pMT-HC-Pro (lane 2) or with pMT-HC-Pro/K (lane 3). Total cell RNA was separated on 1% agarose gels, transferred to nylon membrane and probed with an HC-Pro specific probe. The position of migration of a digoxigenin-labeled RNA molecular weight marker (Roche Molecular Biochemicals) band is shown. C. Northern blot analysis of RNA from DS2-HC-Pro cells (lane 1) or control cells (lane 2). RNA was examined as above. The position of migration of a digoxigenin-labeled RNA molecular weight marker band is shown.

The TEV HC-Pro or CMV 2b proteins were transiently expressed in *Drosophila *DS2 cells and their effect on dsRNA-mediated silencing of a *lac*Z gene examined using a previously described system [20]. Expression of the CMV 2b protein could be detected by immunoblotting in lysates of cells transfected with pMT-2b and induced by addition of CuSO4 to the growth medium (Fig 1A, lane 4). No cross-reacting protein was detected in similarly induced cells not transfected with pMT-2b (Fig 1A, lane 2) or in control cells, or cells transfected with pMT-2b that were not induced (Fig 1A, lanes 1 and 3. In the absence of an antibody to TEV HC-Pro we used an expression plasmid (pMT-HC-Pro/K) that expressed a mutated HC-Pro (TEV K) containing an insertion of 3 amino acids in the central region of the protein [31] as a negative control to verify that any suppressor activity was due to an effect of the HC-Pro protein. This mutant version of HC-Pro was shown previously to be defective in suppression of PTGS in plants [32]. Northern blot analysis of RNA from cells transfected with pMT-HC-Pro or pMT-HC-Pro/K showed that transcript from both plasmids accumulated in the cells (Figure 1B, lanes 2 and 3).

β-galactosidase activity could be detected by staining in ~80% of *Drosophila *cells when they were transfected with a *lacZ *expression plasmid (pMT/V5-His/lacZ) (Table 1). ~17% of cells stained for β-galactosidase when they were co-transfected with pMT/V5-His/lacZ and dsRNA corresponding to the first ~500nt of the *lac*Z gene demonstrating induction of RNAi. No significant decrease in the number of cells stained was observed when cells were transfected with pMT/V5-His/*lacZ *and dsRNA derived from the green fluorescent protein gene indicating that the silencing was specific. Co-transfection of *Drosophila *cells with pMT/V5-His/lacZ, *lacZ*-specific dsRNA and an HC-Pro expression vector (pMT-HC-Pro) resulted in staining of ~46% of cells, indicating that RNAi was being suppressed. Transfection of cells with the mutant pMT-HC-Pro-K along with pMT/V5-His/lacZ and dsRNA resulted in no suppression of RNAi. This indicates that a mutation affecting the ability of HC-Pro to suppress PTGS in plants also affects suppression of RNAi in *Drosophila *cells. Transfection of cells with pMT-2b along with pMT/V5-His/lacZ and dsRNA resulted in no increase in the percentage of transfected cells staining for β-galactosidase activity compared to cells transfected only with pMT/V5-His/lacZ and dsRNA.

### RNAi in stable cell lines expressing TEV HC-Pro or CMV 2b

**Figure 2:**
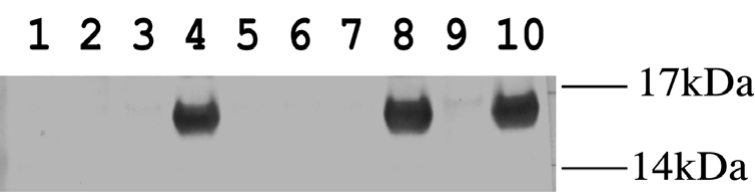
**Expression of TRV 16k protein in *Drosophila *cells. **Immunoblot of lysates from control cells (lanes 1,2 and 5,6), cells transiently transfected with pMT-16k (lanes 3,4) or cells stably transformed with pMT-16k (lanes 7,8). Cells represented in lanes 2, 4, 6 and 8 were induced by addition of CuSO_4 _and cells represented in lanes 1, 3, 5 and 7 were uninduced. Lanes 9 and 10 were lysates from control plant or plant infected with TRV respectively. Lysates were analysed by polyacrylamide gel electrophoresis, proteins transferred to nitrocellulose by electroblotting and probed with an anti-16k antibody. The position of migration of SeeBlue Plus 2 Pre-stained molecular weight markers (Invitrogen) bands is shown.

Stable cell lines expressing HC-Pro (DS2-HC-Pro), or CMV 2b (DS2-2b) were produced in order to improve the efficiency of the RNAi suppression assay by reducing the number of different nucleic acid molecules needed for co-transfection. Immunoblotting of lysates of DS2-2b cells confirmed that the 2b protein accumulated in these cells (Fig 2A, lane 8) and this protein was not produced when the cells were not treated with CuSO4 (Fig 1A, lane 7). Northern blot analysis of RNA from the DS2-HC-Pro cells showed that an HC-Pro-specific transcript accumulated in these cells (Fig 1C, lane 1). An unrelated cell line (DS2-scAb S20) expressing a recombinant antibody [33] (Reavy *et al*., 2000) was used as a control in order to eliminate the possibility that stable transformation of the cells could interfere with the RNAi mechanism. The transfection efficiency of all transformed lines with pMT/V5-His/lacZ was lower than that of the control DS2 cells (Table 1); possibly the transformed cells are more recalcitrant to transfection than the control cells or the additional copies of the metallothionein promoter are saturating the induction factors. Nonetheless, RNAi was strongly induced in the DS2-scAb S20 cells as co-transfection with pMT/V5-His/lacZ and dsRNA reduced the number of cells staining for β-galactosidase activity to only 6.5% compared to ~41% when transfected with pMT/V5-His/lacZ alone (Table 1). Transfection of DS2-HC-Pro cells with pMT/V5-His/lacZ resulted in ~42% of cells expressing β-galactosidase. This was reduced only to ~32% when transfection was carried out using pMT/V5-His/lacZ and dsRNA, showing that RNAi was significantly inhibited in this cell line. In contrast, no suppression of RNAi was observed in the DS2-2b line (expressing the CMV 2b protein) when transfected with pMT/V5-His/lacZ and dsRNA, as the percentage of stained cells was similar to that in DS2-scAb S20 cells transfected with pMT/V5-His/lacZ and dsRNA.

Similar results were obtained when the DS2-2b cells were grown in the presence of CuSO4 to induce expression of the virus protein before transfection with pMT/V5-His/lacZ and dsRNA, indicating that the CMV 2b protein could suppress neither the initiation nor the maintenance of the dsRNA-induced silencing in the *Drosophila *cells.

### Identification of silencing suppression in *Drosophila *cells by another plant virus protein

We were interested to determine if the *Drosophila *cell system could be used as a screen for RNAi suppression effects caused by other virus proteins. We chose to examine the potential RNAi suppression activity of a protein from the tobravirus TRV. This virus is able to suppress transgene silencing in plants [24] but the specific viral protein responsible for this activity has not been identified. TRV, like the other tobraviruses has a bipartite, positive strand RNA genome [34], however, the larger RNA (RNA1) can infect plants systemically in the absence of RNA2 to produce what is known as an NM-infection. This occurs frequently in particular cultivars of potato and is often associated with increased symptom severity. Clearly, therefore, RNA1 encodes all the functions necessary for virus multiplication including, possibly, suppression of PTGS/host defence. The one protein encoded by RNA1 without an assigned function is a 16K cysteine-rich protein which was, thus, investigated as a candidate silencing suppressor protein.

One characteristic of plant virus silencing suppressor proteins is often significant enhancement of disease symptoms when they are over-expressed from a viral vector [26]. Similar results were obtained from preliminary experiments showing that expression of the TRV 16K gene from a PVX vector did increase the severity of symptoms in infected plants. Inoculation of *Nicotiana benthamiana *plants with PVX alone initially induced vein chlorosis and systemic leaf curling, although the plants continued to grow. Inoculation with PVX carrying the 16K gene caused similar initial symptoms but led to tip necrosis and death of the plants [35].

The TRV 16K protein was expressed in *Drosophila *cells after transfection of cells with the expression plasmid pMT-16K, and the 16K protein could be detected by western blotting when the cells were induced with CuSO4 (Fig 2, lane 4) but not when cells were not induced (Fig 2, lane 3). A stably-transformed cell line (DS2-16k) containing pMT-16k behaved in a similar way (Fig 2, lanes 7, 8). No cross-reacting protein was detected in non-transfected cells (Fig 2, lanes 1,2). Co-transfection of pMT/V5-His/lacZ with dsRNA and pMT-16K resulted in ~47% of transfected cells staining blue (Table 1) compared to ~18% when transfected with pMT/V5-His/lacZ and dsRNA. The 16K protein therefore partially suppressed RNAi in *Drosophila *cells.

### Detection of *lacZ *gene transcripts

**Figure 3:**
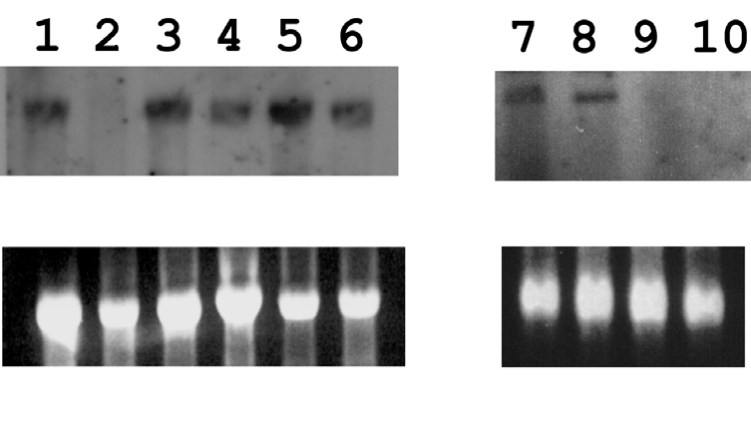
**Northern blot analysis of *lacZ *RNA in DS2 cells. **Upper Panels. Northern blot analysis of total cellular RNA probed with a *lacZ*-specific probe corresponding to ~500 bp of the 5' end of the *lac*Z gene. RNA was extracted from DS2 cells (lanes 1, 2, 7 and 9), DS2-HC-Pro cells (lanes 3 and 4), DS2-16k cells (lanes 5 and 6) or DS2-2b cells (lanes 8 and 10). All cells were transfected with pMT/V5-His/lacZ and lanes 2, 4, 6, 9 and 10 were co-transfected with pMT/V5-His/lacZ and dsRNA. The *lacZ *specific band migrated between 2661nt and 4742 nt digoxigenin labelled RNA size markers. Lower Panels – Stained gels showing loadings of *Drosophila *rRNA before blotting.

Northern blot analysis of the RNA present in transfected cells confirmed that HC-Pro was effective in preventing cytoplasmic degradation of the *lacZ *transcript. RNA with the expected size of the *lacZ *transcript could not be detected in extracts of DS2 cells that were transfected with pMT/V5-His/lacZ and dsRNA (Fig. 3, lane 2). However, intact *lac*Z RNA was present in extracts of DS2-HC-Pro cells that were transfected with pMT/V5-His/lacZ regardless of whether the cells were co-transfected with dsRNA (Fig 3, lanes 3 & 4). Similarly, the *lacZ *transcript was intact in DS2-16K cells after transfection with pMT/V5-His/lacZ and dsRNA (Fig 3, lane 6). The amounts of *lacZ *transcript in the DS2-Hc-Pro and DS2-16K cells were less after transfection with pMT/V5-His/lacZ and dsRNA than after transfection with pMT/V5-His/lacZ alone indicating partial suppression of RNAi. No *lacZ *transcript was detected in DS2-2b cells after transfection with pMT/V5-His/lacZ and dsRNA (Fig 3, lane 10) but the *lacZ *transcript could be detected in DS2-2b cells transfected with pMT/V5-His/lacZ alone Fig 3, lane 8).

### Detection of siRNAs

**Figure 4:**
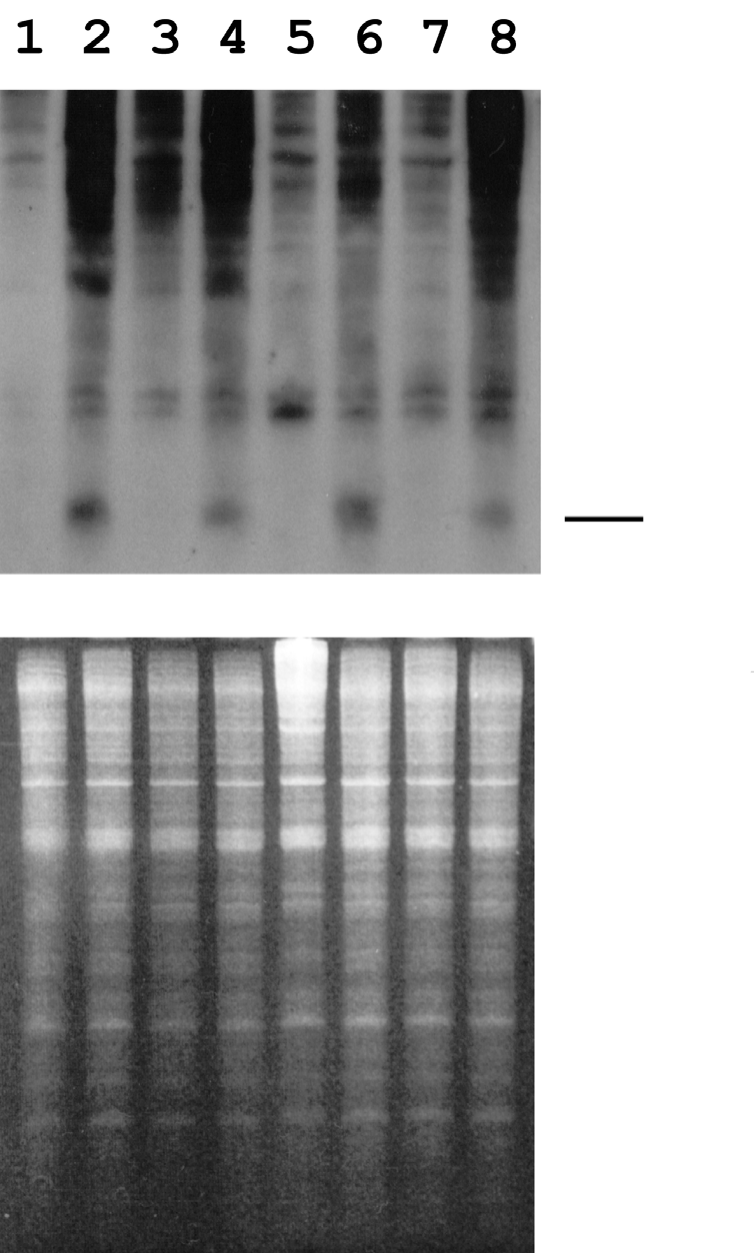
**Detection of small *lacZ *gene-specific RNA. **Total RNA was isolated from *Drosophila *cell cultures and fractionated by chromatography using sepharose CL-2B agarose (Sigma) in Micro Bio-Spin columns (Bio Rad). Fractionated samples were analysed in 15% polyacrylamide-UREA gels and stained with ethidium bromide before the RNA species were transferred to Hybond N+ membrane (Amersham) by electroblotting. The membrane was probed with an antisense digoxigenin-labelled lacZ transcript corresponding to ~500 nt at the 5' end of the *lacZ *gene. The upper panel shows the small RNA species detected by probing with a lacZ gene-specific probe in DS2 cells (lanes 1, 2), DS2-HC-Pro cells (lanes 3, 4), DS2-2b cells (lanes 5, 6) and DS2-16k cells (lanes 7, 8). Cells represented in lanes 1, 3, 5 and 7 were transfected with pMT/V5-His/lacZ and those represented in lanes 2, 4, 6 and 8 were transfected with pMT/V5-His/lacZ and dsRNA. The line to the right indicates the position of GFP specific siRNAs produced in plants as described by Canto et al. (47). The lower panel shows the RNA species detected by staining of the gel before electroblotting.

Suppression of silencing by viral proteins in plants is often associated with an inhibition of the production of small, 21 to 25 nucleotide RNAs that may be analogous to the 22 nucleotide guide RNAs identified as part of the *Drosophila *RISC complex [22,27,36]. We were able to identify short RNAs specific to the region of transfected dsRNA in extracts of *Drosophila *cells transfected with pMT/V5-His/lacZ and dsRNA (Fig 4, lane 2). A lot of larger RNA species were also detected presumably as a result of degradation of the input dsRNA. Unfortunately it is not possible to probe for the presence of short RNAs outwith the region of input dsRNA, as RNAi is not transitive in *Drosophila *[37]. Expression of the plant viral HC-Pro suppressor protein in the DS2-HC-Pro cells did not prevent the accumulation of these small RNAs when transfected with pMT/V5-His/lacZ and dsRNA (Fig 4, lane 4). Co-transfection of *Drosophila *cells with pMT/V5-His/lacZ, dsRNA and pMT-16K also resulted in production of small RNA species (Fig 4, lane 8) even though the 16K protein also partially suppresses silencing in the *Drosophila *cells. Possibly the inefficiency of the transfection procedure and the failure of the suppressors to completely suppress RNAi in these experiments masks any visible effect by the suppressors on the accumulation of these molecules. Similarly, small RNAs were detected in the DS2-2b cells after transfection with pMT/V5-His/lacZ and dsRNA (Fig 4, lane 6).

**Figure 5:**
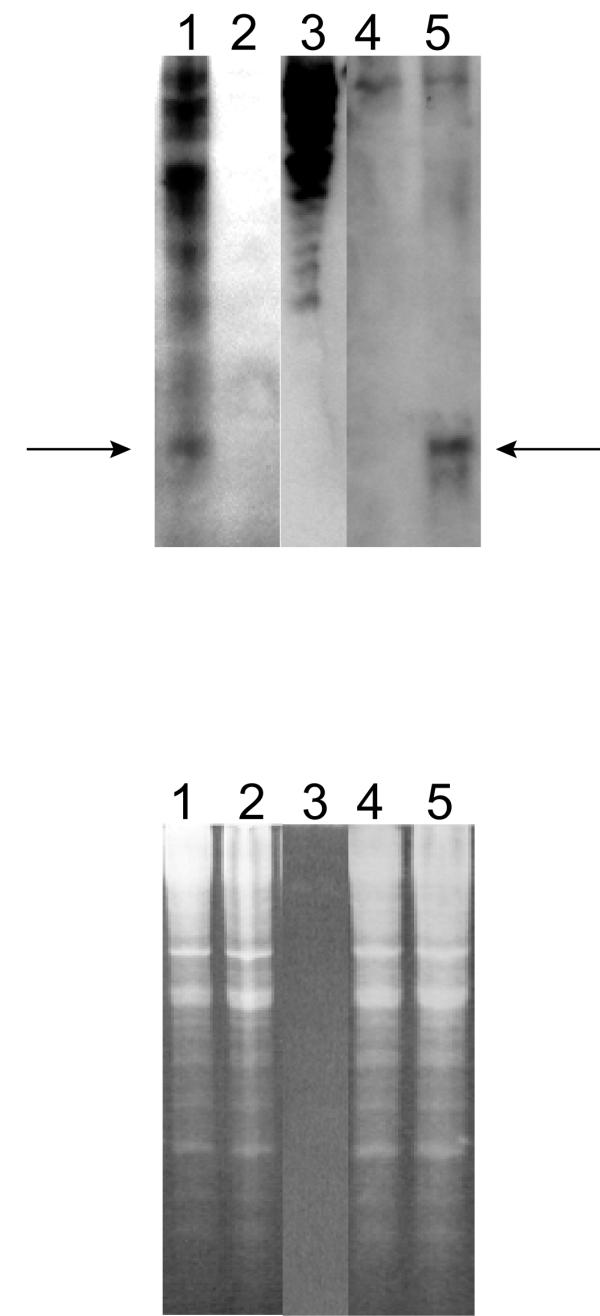
**Small *lacZ *gene-specific RNA is not present in the dsRNA preparation used to induce RNAi. **RNA preparations from *Drosophila *cell cultures were fractionated by chromatography using sepharose CL-2B agarose (Sigma) in Micro Bio-Spin columns (Bio Rad). Fractionated samples were analysed in 15% polyacrylamide-UREA gels, and electroblotted. A digoxigenin-labelled RNA probe complementary to the 5' region of the *lacZ *gene was used for the detection of siRNAs in lanes 1–3. Induction and detection of siRNAs to 35S promoter sequences in agroinfiltrated plant tissue was performed as described by Canto et al. (47) and were used as markers in lanes 4 and 5. The upper panel shows the small RNA species detected by probing with a *lacZ *gene-specific probe in DS2 cells transfected with pMT/V5-His/lacZ (lane 1) or non-transfected DS2 cells (lane 2) or in the dsRNA preparation used to transfect DS2 cells to induce RNAi (lane 3). Also shown are the small RNA species present in plant tissue agroinfiltrated with a binary vector (lane 5) or in non-agroinfiltrated tissue (lane 4) detected using a digoxigenin-labelled RNA probe to 35S promoter sequences present in the binary vector as markers. The positions of migrations of the small (~21nt) RNAs are indicated by arrows.

The dsRNA preparations used to induce RNAi were examined to determine if small RNA species of a similar size to siRNAs were present and were the species detected in figure 4. No short RNA species of 21–25 nucleotides were observed in the dsRNA preparation used to transfect the cells indicating that the species observed in the transfected cells were produced as a result of cellular processing (Fig 5, lane 3). Some larger products were observed and these are likely to have arisen as a result of premature terminations during the dsRNA synthesis reaction.

## Discussion

Suppression of RNAi in *Drosophila *cells by some plant virus proteins indicates that at least part of the processes of RNAi and PTGS is conserved between plants and *Drosophila. *TEV HC-Pro is one of a family of proteins that suppress PTGS in plants and the CMV 2b protein has a similar ability [26]. These proteins are thought to target different components of the PTGS system, as the CMV 2b protein interferes with the spread of a silencing signal after initial induction of silencing, thus, preventing silencing from initiating in newly emerging leaves. The potyvirus HC-Pro protein, however, interferes with the maintenance of silencing in all tissues [26,27]. These differences were reflected in the *Drosophila *cell system where HC-Pro could suppress RNAi but the 2b gene apparently was ineffective. As there is no spread of a silencing signal in cultured *Drosophila *cells, the failure of the CMV 2b protein to act in this system is not unexpected. It was also significant that a mutant version of HC-Pro (K) that is defective in suppression of gene silencing [32] but is effective in proteolytic cleavage [31] was also unable to suppress RNAi in *Drosophila *cells. This strongly supports the idea that the TEV HC-Pro protein targets the same component of the plant PTGS system and the *Drosophila *RNAi system. Our demonstration that the TRV 16K protein also is able to interfere with RNAi in Drosophila cells, leads us to suggest that many other plant virus silencing suppressor proteins are likely to be functional in this system.

SiRNAs were detected in our cell lines expressing HC-Pro and the TRV 16K protein even though suppression of silencing was observed. This is not totally unexpected for a number of possible reasons. Firstly, suppression of RNAi is partial in our cultures indicating that some RNAi, and presumably siRNA production, does occur in some of the cells. We have observed with cells expressing the *lacZ *gene alone that there is considerable variation in the amount of staining of individual cells indicating differences in the amount of gene expression in individual cells. There is no reason to suppose that this variability in gene expression is limited to the *lacZ *gene and it is possible that low levels of the suppressors may fail to inhibit silencing in some of the cells within a culture. Secondly, the experimental protocol requires a time delay between transfection of the cells with the *lacZ *plasmid and dsRNA before induction of expression of the suppressor proteins in order to allow the cells to recover from the transfection procedure. Some production of siRNAs from the dsRNA may possibly occur in cells during this lag period before the suppressors are induced. For these reasons the usefulness of the *Drosophila *RNAi system for studying the mechanisms of action of plant virus suppressor proteins may be limited.

Mutants of *Arabidopsis *that are impaired in PTGS have an increased susceptibility to CMV, showing that PTGS can operate as a defensive system that targets viruses [18]. Plant virus-encoded silencing suppressors are thought to have developed as a response to this defensive aspect of the plant PTGS system, and subsequent studies have shown that, as would be expected, a wide variety of plant viruses encode silencing suppressors [24]. The suppressors encoded by these viruses are unrelated in amino acid sequence, and are likely to act at different points in the silencing process, making them ideal probes to investigate the silencing machinery. We anticipate that using different suppressors from a wide range of viruses may permit a detailed examination of the biochemical process of RNAi in *Drosophila *and possibly other organisms as well as allowing detailed characterisation of the mode of action of the virus suppressor proteins. Furthermore, it is becoming apparent that RNAi or PTGS may play a role in processes other than defence against foreign RNAs. Transformation of plants with TEV HC-Pro or rgs-CaM, a plant-encoded PTGS-suppressor protein related to calmodulin, interrupted normal development and led to the formation of differentiated tumours at the stem/root junction [38]. The *Arabidopsis *gene CARPEL FACTORY is related to *Drosophila *Dicer and is involved in plant development and fertility [39], and the EGO-1 gene of *C. elegans *also appears to have a role in both RNAi and germ-line development 15]. Components of the RNAi mechanism including a homolog of *Dicer *are also involved in synthesis of short temporal RNAs that regulate developmental timing in *C. elegans *[40-42]. Intervention with plant virus silencing suppressors may therefore have significant utility in determining the involvement of RNAi in development and differentiation in plants and animals, and possibly in manipulating these processes.

## Conclusions

These results indicate that at least part of the process of RNAi in *Drosophila *and PTGS in plants is conserved, and that plant virus silencing suppressor proteins may be useful tools to investigate the mechanism of RNAi.

## Methods

### Plasmid constructions

A region (nucleotides 1055–2449) of the TEV genome containing the HC-Pro sequence was amplified by reverse transcription – polymerase chain reaction (RT-PCR) using as a template RNA from an infected plant, and primers HC-Pro-1 (5'-CCGGTACCATGAGCGACAAATCAATCTCTGAGGC-3') and HC-Pro-2 (5'-GGCTCGAGCTACACATCTCGGTTCATCCCTCC-3'). The primers add an ATG codon to the start of the open reading frame and the HC-Pro gene was cloned as a *Kpn*I-*Xho*I fragment into pMT/V5-HisA (Invitrogen) to produce plasmid pMT-HC-Pro. The same primers were also used to clone a mutant HC-Pro gene (TEV-K; [31]) from transgenic plants to produce plasmid pMT-HC-Pro/K. The 2b gene was amplified from RNA2 of CMV isolate Fny using primers 392 (5'-GAACCATGGAATTGAACGTAGGTGC) and 393 (5'-GGGTACCTCAGAAAGCACCTTCCGCC) and cloned into pGemT (Promega) before subcloning into pMT-V5-HisA. The TRV 16K gene was amplified from RNA1 of TRV isolate PpK20 using primers 433 (5'-TCATCATGACGTGTGTACTCAAGGG-3') and 434 (5'-AAGGTACCATCAAAAAGCAAACG-3') to insert *Bsp*HI and *Kpn*I sites upstream and downstream, respectively, of the gene. The PCR product was cloned into pMT/V5-HisA to produce plasmid pMT-16K. The nucleotide sequences of the cloned PCR inserts were confirmed by sequencing.

### dsRNA synthesis

cDNA corresponding to ~500 bp of the 5' end of the *lac*Z gene was amplified using pcDNA3.1/HisB/lacZ (Invitrogen) as a template and primers *lacZ*-1 (5'- TAATACGACTCACTATAGGGAGACCCAAGCTGGCTAGC-3') and *lacZ*-2 (5'- TAATACGACTCACTATAGGGCAAACGGCGGATTGACCG-3'). Both primers contain T7 RNA polymerase promoter sequences (shown underlined). The PCR product was used to direct synthesis of dsRNA using T7 RNA polymerase (Invitrogen) after which the DNA template was removed by DNase digestion.

### Cell culture and transfection

DS2 cells, DES expression medium and the *lacZ *expression plasmid pMT/V5-His/lacZ were supplied as part of the *Drosophila *Expression System (Invitrogen) and cells were grown according to the manufacturer's instructions. Cells were grown in 60 mm dishes and transfected by calcium phosphate co-precipitation with various mixtures of 10 μg each of pMT/V5-His/lacZ and the suppressor expression plasmid DNAs and 5 μg dsRNA. In control transfections, 10 μg of an empty expression plasmid (pMT/V5-HisB) replaced the suppressor expression plasmid. After transfection the cells were washed twice in DES medium and grown for eight hours before expression of proteins was induced by addition of CuSO_4 _to a final concentration of 500 μM. Stably transformed lines expressing the HC-Pro, 2b or a6k genes were established by co-transfection of cells with pMT-HC-Pro or pMT-2b and pCo-Hygro (Invitrogen) followed by selection of transformed cells in medium containing hygromycin. Cells were stained 48 hrs after transfection to detect *lacZ *gene expression using a β-Gal Staining Kit (Invitrogen). Four randomly selected fields of view each containing ~100 cells were selected in each of duplicate plates and the number of cells staining blue was counted.

### Immunoblotting

Cell lysates were analysed by polyacrylamide gel electrophoresis and separated proteins were transferred to nitocellulose using a carbonate buffer [43]. The blots were probed with an anti-CMV 2b [44] or anti 16K antibody [45] followed by a goat anti-rabbit alkaline phosphatase conjugate. Blots were developed using SigmaFast NBT/BCIP substrate (Sigma, Poole, UK).

### Northern blot analysis of RNA from *Drosophila *cells

RNA was extracted from *Drosophila *cells using TriPure Isolation Reagent (Boehringer). Samples of RNA were separated by electrophoresis on formaldehyde/agarose gels, transferred to nylon membrane and probed with digoxigenin-labelled DNA probes corresponding to ~500nts at the 5' end of the *lacZ *gene or to the HC-Pro cDNA described above, as appropriate and essentially as described [46]. The probes were made by PCR using primers lacZ-3 (5'- GGAGACCCAAGCTGGCTAGC-3') and lacZ-4 (5'- GGCAAACGGCGGATTGACCG-3') for the *lacZ *probe, and HC-Pro-1 and HC-Pro-2 for the HC-Pro probe. Digoxigenin-labeled RNA Molecular Weight Marker II (Roche Molecular Biochemicals) was run as a size marker.

For detection of short (~23nt) RNA species total RNA preparations from *Drosophila *cell cultures were fractionated by chromatography using sepharose CL-2B agarose (Sigma) in Micro Bio-Spin columns (Bio Rad), following the manufacturers instructions. ~20 μg of the short RNA species were separated by electrophoresis in a 15% polyacrylamide gel containing 7 M Urea and electroblotted as described by Llave *et al. *[27]. After electrophoresis the gel was stained with ethidium bromide and photographed under ultra-violet light before the RNA species were transferred to Hybond N+ membrane (Amersham) by electroblotting. For the detection of siRNAs, a digoxigenin-labelled RNA probe complementary to the 5' region of the *lacZ *gene as described above was synthesised from a PCR fragment containing the gene fragment downstream of a T7 RNA polymerase promoter, and hybridised to the blots according to the manufacturer instructions (Roche Diagnostics). Induction and detection of siRNAs to GFP or to 35S promoter sequences in agroinfiltrated plant tissue was performed as described by Canto *et al*. [47].

## Competing interests

None declared.

## Authors' contributions

BR conceived of the study, and participated in its design, carried out the *Drosophila *expression experiments, northern blots and drafted the manuscript. SD carried out the immunoassays. TC carried out some of the siRNA assays. SM participated in the design of the study, carried out some of the siRNA assays and contributed to the manuscript. All authors read and approved the final manuscript.

## Figures and Tables

**Table 1:** Effects of plant virus gene silencing suppressors on RNAi in *Drosophila *cells.

Cell Type	Transfection	% cells stained *
DS2	pMT/V5-His/*lacZ*	82.86 ± 9.12
DS2	pMT/V5-His/*lacZ *+ dsRNA	17.70 ± 4.25
DS2	pMT/V5-His/*lacZ *+ dsRNA + pMT-HC-Pro	45.78 ± 4.89
DS2	pMT/V5-His/*lacZ *+ dsRNA + pMT-HC-Pro-K	14.62 ± 2.81
DS2	pMT/V5-His/*lacZ *+ dsRNA + pMT-2b	11.13 ± 1.58
DS2	pMT/V5-His/*lacZ *+ dsRNA + pMT-16K	47.25 ± 3.4
DS2-HC-Pro	pMT/V5-His/*lacZ*	42.04 ± 3.07
DS2-HC-Pro	pMT/V5-His/*lacZ *+ dsRNA	31.70 ± 2.46
DS2-scAb S20	pMT/V5-His/*lacZ*	40.67 ± 2.36
DS2-scAb S20	pMT/V5-His/*lacZ *+ dsRNA	6.50 ± 1.2
DS2-2b	pMT/V5-His/*lacZ*	41.16 ± 2.36
DS2-2b	pMT/V5-His/*lacZ *+ dsRNA	9.17 ± 2.56

* Four randomly selected fields of view each containing ~100 cells were selected in each of duplicate plates and the number of cells staining blue was counted for each experiment. The figures shown are the means from four experiments for the DS2 cells and three experiments for the DS2-HC-Pro and DS2-ScAb S20 cells.
